# Smoking cessation success prediction of parents of children with acute respiratory system disease and influencing factors: a cross-sectional study

**DOI:** 10.1093/fampra/cmag013

**Published:** 2026-04-03

**Authors:** Sema Uçaroğlu, Dilek Menekşe

**Affiliations:** Department of Pediatric Nursing, Institute of Health Sciences, Sakarya University, Sakarya 54050, Türkiye; Child Health of Diseases Clinic, Bursa City Hospital, Bursa 16110, Türkiye; Department of Peadiatric Nursing, Faculty of Health Sciences, Sakarya University, Sakarya 54050, Türkiye

**Keywords:** parents, respiratory tract diseases, smoking, secondhand smoke, smoking cessation

## Abstract

**Background:**

The study aimed to describe the level of smoking cessation success among parents of children with acute respiratory diseases and to examine its associations with selected parental characteristics.

**Methods:**

This analytical cross-sectional study was carried out with 385 smoking parents between November 2022 and July 2023. In the study, the data were collected using the Descriptive Information Form and the Smoking Cessation Success Prediction Scale (SCSPS). The data were analyzed with the SPSS program, using descriptive statistics and multivariable regression analyses. This research was conducted using the convenience sampling approach following the STROBE guideline.

**Result:**

The mean SCSPS score of the parents was 32.65 ± 8.54. Higher SCSPS scores were observed among parents whose children had been hospitalized twice in the past year due to respiratory illnesses (*β* = 0.328, *P* = 0.043) and among parents who had attempted smoking cessation (*β* = 0.321, *P* = 0.001). Moreover, women who tried to quit smoking during pregnancy had significantly higher SCSPS scores (*β* = 0.201, *P* = 0.029). In unadjusted analyses, SCSPS scores differed significantly according to family type and Spouse smoking status (*P* < 0.05). However, this association was not statistically significant in the multivariable regression analysis.

**Conclusion:**

Parents’ smoking cessation success prediction was at a moderate level. Individuals whose children were hospitalized twice in the last year due to respiratory system disease, and who tried to quit smoking, had a higher prediction of smoking cessation success. Moreover, women who tried to quit smoking during pregnancy had a higher prediction of success.

Key messages

- Smoking has significant harmful effects on the parent and child health.
- Smoking and secondhand smoke rates are very high in the world and Türkiye.
- It is known that respiratory system diseases are common in children exposed to secondhand and thirdhand smoke.
- Smoking cessation by parents eliminates children's exposure to secondhand smoke and thirdhand smoke.
- The results showed that the parents’ smoking cessation success prediction was at a moderate level.
- Parents with high smoking cessation prediction should be encouraged and supported to quit smoking.

## Introduction

Smoking and exposure to cigarette smoke are among the greatest threats and epidemics faced by the world, resulting in the illness and death of millions of people, including children [1]. 1.3 billion people in the world and 28.3% of adults in Türkiye regularly smoke tobacco [1,2]. Globally, more than 7 million deaths each year are directly attributable to tobacco smoking, while approximately 1.6 million deaths are caused by passive smoking among nonsmokers [3].

Pediatric SHS exposure constantly and dramatically increases worldwide and affects 40% of children aged 6–19 [4]. Exposure to tobacco smoke has destructive effects throughout childhood and adolescence, starting from pregnancy. This situation mostly affects vulnerable infants and young children since they have smaller and less developed lungs [5, 6]. The World Health Organization and national data have declared respiratory tract infections as the leading cause of mortality and morbidity among newborns and children under five years of age [2, 7]. The current evidence reports that respiratory problems such as pneumonia, bronchiolitis, asthma, dry nocturnal cough, and allergic rhinitis are more common in children living with smokers and exposed to tobacco smoke inside the house [8, 9]. Therefore, some studies have focused on the relationship between cigarette smoke exposure and children's respiratory health [8-10]. A large number of interventions that focus on reducing children's exposure to SHS and thirdhand smoke (THS) inside the house have been developed and shown to be effective. However, the purpose of these interventions has not been to ensure that parents quit smoking but to reduce children's exposure to SHS [9].

Parents’ cessation of smoking is the most effective way to eliminate the harm suffered by young children due to exposure to cigarette smoke inside the house [6]. Thus, parents will also eliminate their own negative health outcomes. Plenty of evidence reports that exposure to SHS and THS may harm the health of infants and children [11, 12]. However, it is a fact that smoking is addictive, and it is not easy to quit nicotine [3].There are many factors that influence parents’ desire to quit smoking, such as self-efficacy, level of addiction, basic emotional variables, concentration difficulties, and withdrawal symptoms. Identifying the motivation of smokers to quit smoking is among the types of interventions recommended in national guidelines [13]. Determining whether parents want to quit smoking and the factors influencing their smoking cessation will be an important step in counseling.

It is important to identify the smoking behaviors of parents of children in the high-risk group with respiratory system disease. Parents of children who visit pediatric clinics should be asked about their smoking habits, informed about the effects of smoking on children, recommended to quit smoking, informed about the helpful medications for smoking cessation, and told that they can participate in smoking cessation programs [12, 14]. To summarize, there is a significant gap between the recommended and the actual implementation. The primary purpose of this study is to determine the smoking cessation success prediction of parents of children with acute respiratory system disease. The secondary purpose is to analytically examine the associations between smoking cessation success estimation and parental sociodemographic and smoking-related characteristics.

## Research questions

1. What is the level of smoking cessation success prediction among parents of children with acute respiratory system disease?
2. Which parental sociodemographic and smoking-related characteristics are associated with smoking cessation success prediction among parents of children with acute respiratory system disease?

## Materials and methods

### Study design

This research was conducted as an analytical cross-sectional study to identify the smoking cessation success prediction of parents of children with acute respiratory disease. The research was carried out in Bursa province in the Marmara Region of Türkiye. It was performed with the parents of children who presented to the hospital between November 2022 and July 2023 and were hospitalized child diseases department and diagnosed with acute respiratory system disease. The Strengthening the Reporting of Observational Studies in Epidemiology (STROBE) statement was used in reporting the article.

### Participants

The study population comprised the smoking parents of children between the ages of 1 month and 5 years, who presented to the hospital with respiratory disease. The study was carried out with 385 parents who met the inclusion criteria, volunteered to participate in the study, and completely filled out the study-related forms. Using the sampling formula in case of unknown population size (*n* = *t*^2^*pq*/*d*^2^), the sample size [*n* = (1.96)^2^ (0.5) (0.5)/(0.05)^2^] was determined as 384. The sampling method used was haphazard sampling. The sampling formula used can be briefly explained as follows; *n*: number of patients sampled, *t*: theoretical value obtained according to the *t*-table at a specific significance level, *p*: frequency of the event under investigation, *q*: frequency of occurrence of the event under investigation, *d*: accepted sampling error according to the event incidence. For a 95% confidence interval, the *t*-value is 1.96, *P* = 0.5, and the accepted sampling error is *d* = 0.05 [15].

**Figure 1:**
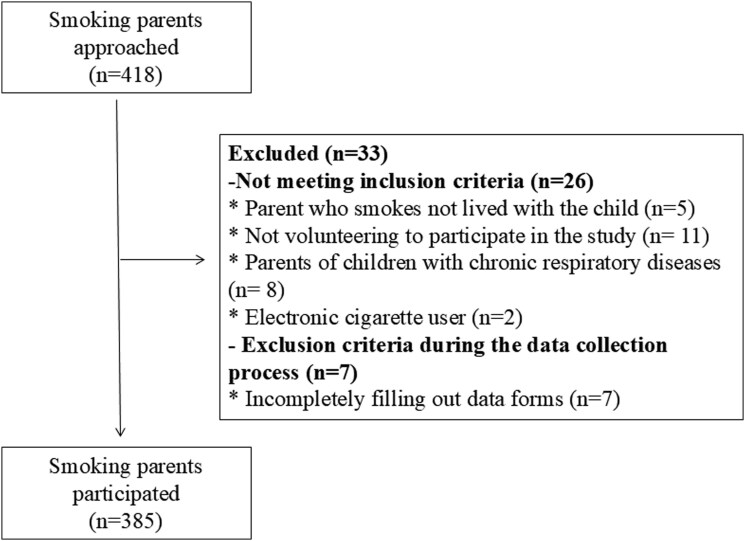
Flow diagram of the study participants.

The inclusion criteria were determined as follows: (a) being a parent over the age of 18, (b) one parent (mother or father) being a smoker, (c) not using electronic cigarettes, (d) being literate, (e) willingness to participate in the study, (f) not having any communication problems, (g) having a child diagnosed with acute respiratory system disease. In the fragmented family type, the smoking parent with whom the child lived was included in the study. Children with rhinitis, acute otitis media, sinusitis, influenza, tonsillitis, pharyngitis, laryngitis, bronchiolitis, and pneumonia are considered among acute respiratory system patients. If both parents of children with acute respiratory disease smoked, only the mother or father was included in the study. Figure 1 shows the reasons regarding the participants who did not meet the inclusion criteria (*n* = 26) and were excluded from the data collection process (*n* = 7) (Fig. 1).

To minimize potential bias, the study population was clearly defined, standard data collection tools were used, and data collectors were trained. The validity and reliability of the measurement tools were considered, and analyses were performed using predetermined methods.

### Variables

#### Independent variables

The descriptive and smoking-related behavioral characteristics of the participating parents were the independent variables of the study. These were: educational level, employment status, economic level, family type, presence of chronic health problems of the participating parents, parental smoking and smoking cessation behaviors, smoking behaviors in the presence of the child, mothers’ smoking and smoking cessation behaviors during pregnancy and breastfeeding, and the frequency of hospitalization of the child due to respiratory system disease.

#### Dependent variables

The dependent variable of the study was the prediction of parental smoking cessation success.

### Data collection tools

#### Descriptive information form

The descriptive information form was prepared by the researchers to determine the descriptive and smoking-related behavioral characteristics of the parents in line with the literature on the subject [16-18].

#### Smoking cessation success prediction scale (SCSPS)

The 5-point Likert (Very Low, Few, Average, Many, Too Many) scale developed by Aydemir et al. (2019) consists of 10 items [16]. The scale has two subscales: Steadiness and Readiness (1,2,6,8,9,10) and Health Perception and Appropriate Environment (3,4,5,7). The highest score that can be received from the scale without any negative items is 50, and the lowest score is 10. The increase in the score shows that the success of smoking cessation will be high. In the original study, the variance of the scale is 50.39%. Cronbach's alpha value of the scale was calculated as 0.78 [16]. In this study, Cronbach's alpha value of the scale was found to be 0.80.

### Data collection

Parents were informed about the purpose of the study. The child was diagnosed with acute respiratory system disease and the parents were interviewed after child was admitted to the child health and diseases department. In the hospital, data were collected in patient rooms of child health and diseases. Data collection forms were given to the participant and asked to fill them out. Parents filled out the form while their children were asleep or during visitor time while the other parent was caring for the child. Filling out the scales took about 10–15 min. The forms were received from the participant after filling them out.

### Data analysis

Statistical analyses were performed using the Windows-based Statistical Package for the Social Sciences (SPSS) 22.0 package software. Number, percentage, arithmetic mean, standard deviation were used for descriptive analyses. The Kolmogorov–Smirnov test was used to examine the normal distribution of the data. An independent sample *t*-test was used in paired groups and a one-way ANOVA test was used in groups of more than two in normally distributed data. Pearson's correlation and regression analyses were conducted to compare variables. Cronbach's alpha value was used in the reliability analysis of the scale. The significance value for all statistics was accepted as *P* < 0.05.

### Ethical considerations

Ethical approval was obtained from the Health Ethics Committee of Bursa City Hospital, Bursa, Türkiye (Date: 20.10.2022 Number: E-13012450-514.99). Institutional permission was received from the hospital where the study was conducted. Permission was acquired from the authors via e-mail to use the SCSPS. The participants were assured that all their personal information would remain confidential. The participants’ verbal and written consents were obtained in line with the principle of volunteering.

## Results

A total of 385 parents were included in the final analysis of the study. Of the parents participating in the study, 33% were mothers and 67% were fathers. Half of the children (54.0%) were diagnosed with lower respiratory tract infection and 46% with upper respiratory tract infection. Some descriptive characteristics and relationship are presented in Table 1.

**Table 1:** Differences in SCSPS and subscale scores by parental descriptive characteristics (*n* = 385).

Children's descriptive characteristics	*n*	%	SCSPS		
			Steadiness and readiness	Health perception and appropriate environment	Total
			Descriptives	Descriptives	Descriptives
**Gender**					
Female	155	40.3	18.56 ± 5.60	14.43 ± 4.16	33.00 ± 8.93
Male	230	59.7	18.35 ± 5.21	14.05 ± 3.90	32.40 ± 8.28
***t*/*P***			0.386/0.700	0.901/0.368	0.665/0.506
**Diagnosis**					
Upper respiratory tract infection	177	46.0	18.74 ± 5.36	14.46 ± 4.10	33.21 ± 8.50
Lower respiratory tract infection	208	54.0	18.17 ± 5.37	13.98 ± 3.92	32.16 ± 8.57
**t/*P***			1.035/0.302	1.179/0.239	1.204/0.229
**Frequency of the child's hospitalization due to respiratory system disease in the last year**					
1 (1)	308	80.0	18.51 ± 5.38	14.31 ± 4.01	32.83 ± 8.55
2 (2)	54	14.0	18.94 ± 5.06	14.61 ± 3.57	33.55 ± 8.01
3 and above (3)	23	6.0	16.17 ± 5.52	11.82 ± 4.34	28.00 ± 8.63
***F*/*P***			2.337/0.098	4.516/0.012* **1** **>** **3**^a^**, 2** **>** **3**^a^	3.834/0.022* **1** **>** **3**^a^**, 2** **>** **3**^a^
**Parent's identifying characteristics**					
**Parent's closeness to child**					
Mother	127	33.0	18.07 ± 5.41	13.89 ± 4.33	31.97 ± 9.04
Father	258	67.0	18.61 ± 5.34	14.36 ± 3.84	32.97 ± 8.28
***t*/*P***			−0.923/0.356	−1.064/0.288	−1.080/0.281
**Education**					
Primary school	134	34.8	18.16 ± 5.35	13.97 ± 4.26	32.13 ± 8.78
High school	150	39.0	18.81 ± 5.54	14.38 ± 4.07	33.20 ± 8.77
University and above^b^	101	26.2	18.24 ± 5.12	14.25 ± 3.57	32.50 ± 7.90
***F*/*P***			0.603/0.548	0.391/0.677	0.568/0.567
**Employment status**					
Employed	273	70.9	18.70 ± 5.40	14.45 ± 3.79	33.16 ± 8.31
Unemployed	112	29.1	17.79 ± 5.24	13.59 ± 4.46	31.39 ± 9.01
***t*/*P***			1.511/0.132	1.916/0.056	1.849/0.065
**Economic status (according to his/her own statement)**					
Income is less than my expenses	123	31.9	18.11 ± 5.72	14.26 ± 3.91	32.38 ± 8.69
Income is equal to my expenses	216	56.1	18.42 ± 5.06	13.92 ± 4.06	32.34 ± 8.42
Income is more than my expenses	46	11.9	19.39 ± 5.77	15.39 ± 3.85	34.78 ± 8.59
***F*/*P***			0.950/0.388	2.587/0.077	1.637/0.196
**Family type**					
Nuclear family (1)	324	84.2	18.76 ± 5.32	14.33 ± 4.02	33.09 ± 8.48
Extended family (2)	44	11.4	16.11 ± 4.60	13.15 ± 3.73	29.27 ± 7.71
Broken family (3)	17	4.4	18.23 ± 6.70	14.58 ± 4.33	32.82 ± 10.18
***F*/*P***			4.833/0.008** **1** **>** **2**^a^**, 3** **>** **2**^a^	1.737/0.177	3.938/0.020* **1** **>** **2**^a^**, 3** **>** **2**^a^
**Number of living children**					
1	140	36.4	18.57 ± 4.97	14.07 ± 3.74	32.65 ± 7.86
2	166	43.1	18.75 ± 5.47	14.50 ± 4.08	33.25 ± 8.65
3 and above	79	20.5	17.53 ± 5.77	13.83 ± 4.32	31.36 ± 9.39
***F*/*P***			1.463/0.233	0.861/0.424	1.035/0.272
**Chronic health problem**					
Yes	27	7.0	17.03 ± 4.56	14.14 ± 3.95	31.18 ± 7.53
No	358	93.0	18.54 ± 5.41	14.21 ± 4.02	32.75 ± 8.61
***t*/*P***			−1.409/0.160	−0.080/0.936	−0.921/0.358

Bold values indicate statistical significance.

Upper respiratory tract infection: influenza (*n* = 60), pharyngitis (*n* = 48), tonsillitis (*n* = 69); lower respiratory tract infection: bronchiolitis (*n* = 56), bronchitis (*n* = 51), bronchopneumonia (*n* = 44), pneumonia (*n* = 57).

^a^Tukey Anova Test.

^b^Postgraduate education *n* = 2 (%0.5).

*P < 0.05; **P < 0.01.

SCSPS, Smoking Cessation Success Prediction Scale.

*F* = one-way Anova.

As seen in Table 2, the parents whose spouses smoked were determined to have a significantly lower smoking cessation success prediction compared to the parents whose spouses did not smoke (*P* < 0.05). The smoking cessation success predictions of those who tried to quit smoking in the past and those who tried to quit smoking during pregnancy were significantly higher compared to those who did not try (*P* < 0.05). 9.6% of the parents reported that they could quit smoking today, 3.6% in the next week, 5.5% in the next month, 6.0% in a year, and 75.3% had no idea when they would quit. It was determined that the SCSPS total mean score of parents who had no idea when to quit smoking was 30.58 and was significantly lower than the other groups (*P* < 0.05).

**Table 2:** Differences in SCSPS and subscale scores by parental smoking behavior (*n* = 385).

Parents’ smoking behaviors	*n*	%	SCSPS		
			Steadiness and readiness	Health perception and appropriate environment	Total
			Descriptives	Descriptives	Descriptives
**Frequency of smoking per day (pcs)**					
1–10	177	46.0	18.89 ± 5.12	14.10 ± 4.03	33.00 ± 8.37
11–20	187	48.6	17.98 ± 5.65	14.28 ± 4.01	32.27 ± 8.80
21 and above	21	5.5	18.57 ± 4.68	14.38 ± 3.90	32.95 ± 7.89
***F*/*P***			1.312/0.270	0.108/0.898	0.347/0.707
**Spouse smoking status**					
Yes	157	40.8	17.85 ± 5.31	13.69 ± 4.23	31.55 ± 8.86
No	228	59.2	18.83 ± 5.37	14.56 ± 3.82	33.39 ± 8.25
***t*/*P***			−1.761/0.079	−2.093/0.037*	−2.090/0.037*
**Presence of someone else smoking at home other than their spouse**					
Yes	52	13.5	17.57± 5.27	14.01± 3.77	31.59± 8.15
No	333	86.5	18.57± 5.37	14.23± 4.05	32.81± 8.60
***t*/*P***			−1.246/0.214	−0.364/0.716	−0.953/0.341
**Smelling tobacco in the house**					
Yes	91	23.6	17.89 ± 5.51	13.93 ± 4.16	31.82 ± 8.72
No	294	76.4	18.60 ± 5.32	14.29 ± 3.96	32.90 ± 8.49
**t/*P***			1.096/0.275	0.744/0.457	1.036/0.302
**Smoking near the child**					
Yes	28	7.3	19.89 ± 6.16	14.07 ± 4.65	33.96 ± 10.13
No	357	92.7	18.32 ± 5.29	14.21 ± 3.96	32.54 ± 8.41
**t/*P***			1.490/0.137	−0.187/0.852	0.847/0.398
**Trying to quit smoking in the past**					
Yes^a^	274	71.2	18.93 ± 5.37	14.72 ± 3.96	33.65 ± 8.45
No	111	28.8	17.22 ± 5.19	12.93 ± 3.85	30.16 ± 8.30
***t*/*P***			2.849/0.005**	4.034/0.000***	3.689/0.000***
**Time period when think about quitting smoking**					
I can quit smoking today (1)	37	9.6	23.29 ± 5.00	16.10 ± 3.77	39.40 ± 7.79
In the next week (2)	14	3.6	24.50 ± 3.48	17.28 ± 2.39	41.78 ± 4.66
In the next month (3)	21	5.5	22.09 ± 5.85	16.33 ± 4.76	38.42 ± 10.37
After one year (4)	23	6.0	21.43 ± 4.37	15.52 ± 3.48	36.95 ± 6.42
I have no idea when to quit (5)	290	75.3	17.02 ± 4.72	13.55 ± 3.87	30.58 ± 7.73
***F*/*P***			27.080/0.000*** **5** **>** **1,2,3,4^b^**	27.080/0.000*** **5** **>** **1,2,3^b^**	21.737/0.000*** **5** **>** **1,2,3,4^b^**
**Mothers’ smoking behaviors during pregnancy and breastfeeding**					
**Smoking status during pregnancy (*n*** **=** **127)**					
Yes	53	41.7	16.94 ± 5.14	13.33 ± 4.47	30.27 ± 8.96
No	74	58.3	19.06 ± 5.55	14.36 ± 4.20	33.42 ± 9.05
***t*/*P***			−2.206/. 029*	−1.331/0.186	−1.957/0.053
**Trying to quit smoking during pregnancy (*n*** **=** **127)**					
Yes	96	75.6	18.61 ± 5.49	14.42 ± 4.11	33.04 ± 8.93
No	31	24.4	16.38 ± 4.83	12.22 ± 4.49	28.61 ± 8.56
***t*/*P***			2.017/0.046*	2.529/0.013*	2.424/0.017*
**Smoking status during breastfeeding (*n*** **=** **127)**					
Yes	83	65.4	17.75 ± 5.60	13.42 ± 4.40	31.18 ± 9.25
No	44	34.6	18.70 ± 5.11	14.86 ± 4.08	33.56 ± 8.65
***t*/*P***			−0.931/0.354	−1.801/0.074	−1.414/0.160

Bold values indicate statistical significance.

SCSPS, Smoking Cessation Success Prediction Scale; *F* = One-Way Anova, *t* = independent sample *t* test.

*P < 0.05 **P < 0.01 ***P < 0.001.

^a^Parents who have tried to quit smoking in the past; getting professional support (8.6%), reducing self-smoking (52.2%) and chewing nicotine gum (2.3%). More than one mark was made in the answers.

Table 3 presents the correlations between selected descriptive characteristics and the total and subscale scores of the SCSPS. A statistically significant positive correlation was identified between the number of attempts to quit smoking and the mean total score of the SCSPS (r = 0.236 *P* = 0.000). (Table 3).

**Table 3:** Correlations between selected descriptive characteristics and the total and subscale scores of the SCSPS (*n* = 385).

Descriptive characteristics	Mean ± SD	Min	Max	Steadiness and readiness	Health perception and appropriate environment	SCSPS total
				*r*/*P*	*r*/*P*	*r*/*P*
Child age (Month)	29.31 ± 2.32	1	60	−0.038/0.454	0.014/0.791	−0.018/0.730
Parent's age (year)	33.24 ± 6.64	19	59	−0.005/0.916	0.095/0.062	0.041/0.420
Frequency of smoking per day (pcs)	13.76 ± 8.36	1	40	−0.075/0.140	0.042/0.408	−0.027/0.591
Smoking duration	13.05 ± 7.06	1	34	−0.030/0.563	0.119/0.019*	0.037/0.464
Number of attempts to quit smoking	2.39 ± 2.42	1	30	0.234/0.000***	0.185/0.002**	0.236/0.000***
The effect of the child on wanting to quit smoking^a^	5.72 ± 3.72	0	10	0.072/0.241	0.135/0.026*	0.109/0.073
Frequency of smoking during pregnancy (pcs/day)	6.13 ± 5.75	1	20	−0.012/0.930	0.154/0.265	0.070/0.614
Steadiness and Readiness	18.43 ± 5.36	6	30		0.653/0.000***	0.935/0.000***
Health perception and appropriate environment	14.230 ± 4.01	4	20			0.880/0.000***
SCSPS Total	32.65 ± 8.54	12	50			

Bold values indicate statistical significance.

*P < 0.05 **P < 0.01 ***P < 0.001.

^a^This explains the child's influence on the parent's desire to quit smoking (0: no efficacy 10: very much efficacy). Answers are based on parents’ self-report.

Table 4 shows that the regression model developed to predict smoking cessation success was statistically significant (*F* = 3.165, *P* = 0.004). In the model, the variables of the child's number of hospitalizations, trying to quit smoking during pregnancy, and number of trying to quit smoking were found to be significant. Parents whose child was hospitalized two times due to acute respiratory disease have a higher score than those whose child was hospitalized three or more times (*β* = 0.328 *P* = 0.043). As the number of parents’ attempts to quit smoking increases, smoking cessation success prediction scores increase (*β* = 0.321, *P* = 0.001). Those who quit smoking during pregnancy have a higher SCSPS score than those who did not quit smoking during pregnancy (*β* = 0.201, *P* = 0.029). In the established model, 11.9% of the SCSPS scores were explained by independent variables (Adj. *R*^2^ = 0.119). Additionally, there is no multicollinearity problem (VIF < 10) and autocorrelation problem (DW = 1.681) in the model (Table 4).

**Table 4:** Regression analysis of selected descriptive characteristics predicting smoking cessation success.

Model 1 variables		SCSPS					
		*B*	SE	*β*	*t*	*P*	VIF
Constant		18.009	4.506		3.997	0.000	
Number of attempts to quit smoking		1.738	0.495	0.321	3.510	**0**.001**	1.064
Smoking status during pregnancy (reference no)							
Smoking status during pregnancy	Yes	5.022	2.262	0.201	2.220	**0**.029*	1.038
Frequency of the child's hospitalization due to respiratory system disease in the last year (reference 3 and above)							
Frequency of the child's hospitalization due to respiratory system disease in the last year	1	6.143	3.913	0.251	1.570	0.119	3.253
	2	9.214	4.497	0.328	2.049	**0**.043*	3.266
Family type (reference nuclear family)							
Family type	Extended family	−2.121	2.860	−0.067	−0.742	0.460	1.047
	Broken family	1.954	2.864	0.062	0.682	0.497	1.050
Spouse smoking status (reference no)							
Spouse smoking status	Yes	0.537	1.959	0.025	0.274	0.784	1.079
**F** **=** **3.165; *P*** **=** **0.004; DW** **=** **1.681; Adj. R^2^** **=** **0.119**							

Bold values indicate statistical significance.

*B*, unstandardized beta; SE, standard error; *β*, standardized beta *β*; *R*, correlation; *R*2, correlation coefficient (explained variance ratio); *F*, model statistics; *P*, level of significance; VIF, variance inflation factor.

*P < 0.05 **P < 0.01.

## Discussion

Health behavior theories emphasize the importance of cognitive and environmental determinants of behavior. The individual's desire and determination for quitting smoking determine his/her prediction [16]. The mean total score of the SCSPS is 32.65 ± 8.54. This mean score indicates a moderate level. Another study using the scale stated that the mean total score of smoking cessation success prediction in the pregnancy plans of women who smoked was 31.51 ± 8.17 [18]. The similarity between the results of both studies can be explained by the fact that the studies were conducted in the same country and similar culture. Although the SCSPS score is relatively good in our sample, as emphasized in many studies, the need for support and counseling assistance services, which will increase parents’ self-confidence and lead them toward smoking cessation, is evident [14, 19].

Of the parents, 71.2% tried to quit smoking in the past. Of the parents, 8.6% tried to quit by receiving professional support, 52.3% by reducing smoking on their own, and 2.3% by chewing nicotine gum. Although a high proportion of parents had previously attempted to quit smoking, many continued to smoke, indicating challenges in achieving sustained smoking cessation. Similar to our study, recent studies have found the rates of individuals who try to quit smoking as 66.2% [3] and 68% [20]. Interventions such as training, motivational interviewing, support, and counseling by health professionals enhance smoking cessation success [19, 21]. These results indicate the need for professional support to encourage and guide parents for smoking cessation.

The SCSPS scores of parents who tried to quit smoking in the past were found to be higher. Likewise, it was revealed that women who quit smoking in their previous pregnancies had a higher smoking cessation success prediction [18]. When the literature is examined, it is reported that the number of quit attempts is important for smokers to be successful in quitting smoking. Chaiton et al. emphasized that understanding that many smokers may require 30 or more quit attempts to be successful may help clinical prospects [22]. These results may be linked to individuals’ learning by experiencing the process and eliminating uncertainties.

One of our main findings is that, in the study, the smoking cessation success prediction of parents whose children were hospitalized three and more times due to respiratory system disease in the last year was significantly lower compared to parents of children who were hospitalized one and two times. Many studies have reported that parents’ smoking increases the prevalence of respiratory system diseases in their children [10, 14]. For this reason, it is emphasized that hospitalization rates are significantly higher [4, 11]. Despite the negative effects of smoking on their own and their children's health, the SCSPS low score of parents whose children are hospitalized three times or more may be related to and number of attempts to quit smoking.

In our study, unfortunately, 7.3% stated that they smoked near the child. This rate cannot be underestimated. Although SHS awareness studies continue to be conducted increasingly, the rates are reported to be high, between 14.4% and 99.2% [10, 21, 23]. Furthermore, considering the possibility of exposure to THS, which could not be determined in the study, the results emphasize the need for protecting children against exposure to their parents’ smoking. These results indicate inadequate parental knowledge, attitude, and practice regarding environmental tobacco smoke.

Our study determined that the rate of smoking among women was 41.7% during pregnancy and 65.4% during breastfeeding. Previous studies have stated the prevalence of smoking during pregnancy as 21% and 20–24%[17, 24]. It has been reported in various studies that the adverse effect of smoking during pregnancy on pregnancy and the infant (miscarriage, stillbirth, prematurity, low birth weight, intrauterine growth restriction, congenital abnormalities, and neonatal or sudden infant death) is an important predictor for parents to quit smoking [25, 26]. In another study, knowing that smoking during pregnancy had a negative impact on pregnancy and the infant positively affected the SCSPS [18]. Pregnancy has been described as a life event that strongly motivates women to quit smoking [27]. This period may be an opportunity for individuals to quit smoking. A remarkable point in our findings was that mothers who quit smoking during pregnancy started smoking again during breastfeeding. It is similar in the literature [27-29]. Another study reported that women who quit smoking during pregnancy started smoking behavior two months after birth [30]. It is known that the active substances of cigarettes are transferred through breast milk and adversely affect the infant's health [31]. The smoking cessation success prediction of those who tried to quit smoking during pregnancy was significantly higher.

The study has some limitations. The data were collected from a single center (hospital) and cannot be generalized. The measured cases are limited to the scale items, and the scale implementation is based on self-report. Parents’ smoking addiction levels were not measured. A significant limitation of the study is that mothers tend to underreport their smoking habits due to concerns about social acceptability. It is recommended that these factors be considered in interpreting the findings. The study's strength is that the sample group included both mothers and fathers of children. Given the literature [32] indicating that fathers have higher smoking rates compared to mothers, the fact that two-thirds of the sample consists of fathers exhibits a distribution consistent with the smoking patterns described in the literature. Furthermore, the sufficient sample size increased the ability of the findings to represent the population.

## Conclusion

The study results show that the parents’ SCSPS scores are at a moderate level. Individuals whose children were hospitalized twice times in the last year due to respiratory system disease, and who tried to quit smoking had a higher prediction of smoking cessation success. Moreover, women who tried to quit smoking during pregnancy had a higher prediction of smoking cessation success. Moreover, it is necessary to take into account the variables that may be effective in parents’ quitting smoking. Nurses can encourage and support parents with high smoking cessation predictions to quit smoking. The results of the present study will provide some data on recognizing the obstacles for parents to quit smoking. Especially determining and addressing the factors that influence smoking cessation will increase success predictions. It is recommended to conduct interventional randomized controlled and longitudinal studies by identifying individuals with high smoking cessation potential in future research. Studies can be conducted to evaluate the relationship between smoking addiction and smoking cessation success prediction. Furthermore, it is recommended to form standardization by developing national diagnostic guidelines involving the variables that affect smoking cessation.

## Data Availability

The data files are available on request from the authors.
